# Postpartum Rapid Cardiopulmonary Deterioration in an Obese Mother and Congenital Pneumonia in the Neonate: A Dual Case Report

**DOI:** 10.7759/cureus.86735

**Published:** 2025-06-25

**Authors:** Varuni Karnasula, Jaya L Dasari

**Affiliations:** 1 General Practice, Rohan Hospital, Hyderabad, IND; 2 Obstetrics and Gynecology, Rohan Hospital, Hyderabad, IND

**Keywords:** cardiac dysfunction, congenital pneumonia, maternal obesity, neonatal sepsis, pneumonia, postpartum complications

## Abstract

The postpartum period demands close monitoring due to rapid hemodynamic and hormonal changes that can unmask subclinical conditions or exacerbate mild illnesses, particularly in individuals with comorbidities such as obesity. We report a case of a 31-year-old woman at 36 weeks of gestation with obesity and hypothyroidism, who presented with mild respiratory symptoms and had grade I diastolic dysfunction. Although outpatient treatment was initiated for a suspected respiratory infection, multidisciplinary evaluation led to the decision for cesarean delivery under spinal anesthesia. A late preterm female neonate was delivered, who subsequently developed respiratory distress, diagnosed as congenital pneumonia, and was managed successfully. Postoperatively, the mother’s respiratory symptoms worsened, diastolic dysfunction progressed, and imaging revealed bilateral pleural effusion with right lung opacities, accompanied by elevated levels of C-reactive protein (CRP), an inflammatory marker. She was managed with appropriate antimicrobial therapy and supportive care, which resulted in gradual clinical improvement. This case report highlights the need for antepartum risk stratification, timely intervention, and meticulous postpartum monitoring to prevent adverse maternal and neonatal outcomes.

## Introduction

Pregnancy and obesity are independently associated with immunosuppression and altered respiratory physiology. During pregnancy, maternal cell-mediated immunity is downregulated to promote fetal tolerance, increasing susceptibility to infections [1]. Respiratory physiologic changes in pregnancy include increased oxygen consumption, reduced chest wall compliance, diaphragmatic elevation, and a decrease in functional residual capacity (FRC) [2]. Obesity constitutes a chronic low-grade inflammatory state that impairs the host's immune defenses [3]. It further compromises respiratory mechanics by reducing chest wall compliance, lowering FRC and expiratory reserve volume (ERV), and increasing airway resistance, resulting in ventilation-perfusion mismatch [3]. A 2023 randomized study linked obesity to a higher risk of respiratory infections, with an odds ratio of 1.17 for pneumonia and 1.303 for unspecified lower respiratory tract infections (LTRIs) [4]. These effects of pregnancy and obesity may act synergistically, particularly in the third trimester when alterations in respiratory mechanics are most pronounced, thereby significantly increasing the risk of respiratory infections.

The postpartum period requires high vigilance as it accounts for increased maternal morbidity and mortality. A systematic review in 2022 reported that approximately 73% of the maternal deaths occur within the first week of postpartum [5]. This dual case report illustrates rapid postpartum deterioration in the mother despite the absence of overt warning signs, likely driven by abrupt postpartum physiological shifts compounded by obesity-related respiratory compromise with concomitant respiratory infection. It emphasizes the need for careful observation and timely maternal intervention during the early postpartum period, particularly in high-risk patients. Furthermore, preparedness for anticipated neonatal complications, especially when maternal perinatal infection raises concern for vertical transmission, can help prevent adverse outcomes, as demonstrated in our case.

## Case presentation

A 31-year-old gravida 2, para 1 woman at 36 weeks gestation presented to the outpatient department with a two-day history of subjective fever, intermittent dry cough, and mild dyspnea. She denied recent travel or contact with individuals exhibiting similar symptoms.

On examination, the patient weighed 80 kg, with a height of 140 cm, corresponding to a body mass index (BMI) of 40.8 kg/m^2^. Her pre-pregnancy BMI was 36.7 kg/m^2^, classified as grade II obesity according to the World Health Organization (WHO) classification (35-39.9 kg/m^2^). 

Her vital signs included a blood pressure of 110/70 mm Hg, pulse rate of 130 beats per minute with a regular rhythm, respiratory rate of 18 breaths per minute, oxygen saturation of 97% on room air, and a temperature of 100°F. Chest auscultation was clear. Cardiotocography showed fetal tachycardia with a heart rate ranging from 160 to 180 beats per minute. Echocardiography revealed grade I diastolic dysfunction. The levels of C-reactive protein (CRP) were mildly elevated at 7.03 mg/dL, suggesting an early inflammatory response.

The patient had hypothyroidism, well managed with thyroid hormone supplementation. Her antenatal course was uneventful with the exception of a urinary tract infection at 26 weeks of gestation. Urine culture identified *Klebsiella *species, which were successfully treated with oral antibiotics. She remained normotensive and had no evidence of preeclampsia or gestational diabetes. Antenatal corticosteroids were not administered, as there were no signs of preterm labor or other indications for early delivery. She was a non-smoker, did not consume alcohol, and had no history of illicit drug use. During her previous pregnancy three years prior, she developed preeclampsia at 33 weeks of gestation, managed by labetalol and lifestyle modifications, and delivery was via caesarean section at 37 weeks and was normotensive in the postpartum period.

The potential risks of continuing the current pregnancy for both the mother and fetus were discussed with input from the obstetrician, general physician, and pediatrician, considering the mother's worsening subjective dyspnea and persistent fetal tachycardia, which raised concerns about possible intrauterine infection and risk of fetal compromise or loss. A joint multidisciplinary decision was made to perform lower-segment cesarean section (LSCS) under spinal anesthesia, which was consented to by the patient. She was initiated on parenteral antibiotics and underwent uneventful surgery.

She delivered a late preterm female neonate weighing 2.5 kg with appearance, pulse, grimace, activity, and respiration (APGAR) scores of 7 and 9 at one and five minutes, respectively. No congenital external anomalies were noted. Within 15-20 minutes of birth, the neonate developed tachypnea with 82 breaths per minute, tachycardia with 192 beats per minute, and oxygen desaturation with SpO_2_ ranging from 82 to 86% at room air. The neonate was started on low-flow oxygen. The neonate’s initial blood sugar levels was 33 mg/dL, corrected with intravenous 5% dextrose; it subsequently increased to 64 mg/dL. The neonate exhibited nasal flaring, grunting, and subcostal retractions with a Downes score of 6 out of 10. In view of the increased requirement of respiratory support, the neonate was started on non-invasive positive pressure ventilation (NIPPV), empirical parenteral antibiotics, piperacillin-tazobactam, and amikacin, which were initiated and continued for five days. Chest radiograph showed bilateral lung infiltrates. Blood cultures showed no bacterial growth. Placental tissue sent for culture and Gram staining also showed no growth.

The neonate was initially kept nil per os (NPO), followed by the introduction of orogastric feeds, which were gradually increased and subsequently transitioned to oral feeding by the third day of life. The neonate tolerated the feeds well, with no complications. After 44 hours of NIPPV, it was gradually weaned to venturi-based continuous positive airway pressure (CPAP) for 20 hours, followed by high-flow nasal cannula (HFNC) for eight hours, and eventually weaned to room air.

Maternal postoperative course

On postoperative day 1, the patient reported worsening of her cough, now productive with whitish sputum, dyspnea, and orthopnea.

Although her vitals remained stable with blood pressure of 110/70 mm Hg, pulse rate of 78 beats per minute, respiratory rate of 16 breaths per minute, temperature of 98°F, and oxygen saturation of 95-96% on room air, chest auscultation revealed bilateral wheezing, more pronounced on right side, along with bilateral crepitations. Chest radiograph showed bilateral pleural effusions, patchy opacities in the right middle lobe and bilateral basal lobes, and mild cardiomegaly. Repeat echocardiography revealed persistent grade I diastolic dysfunction with mild tricuspid regurgitation (TR), and mild pulmonary arterial hypertension (PAH). CRP levels were markedly elevated to 63 mg/dL, indicating progression of the inflammation.

She was empirically started on piperacillin-tazobactam, azithromycin, oseltamivir, low-dose furosemide to alleviate pleural effusion, nebulized with salbutamol for bronchodilation, and enoxaparin for thromboprophylaxis.

On postoperative day 2, respiratory distress worsened, with a respiratory rate of 24 breaths per minute, and oxygen saturation dropped to 88% on room air. She was started on low-flow oxygen, encouraged to frequent ambulation, and commenced on chest physiotherapy. Chest computed tomography (CT) showed focal consolidation in bilateral basal segments and the right middle lobe lateral segment. Repeat echocardiography revealed progression to grade II diastolic dysfunction, persistent mild TR, and mild PAH. Influenza panel, blood, and urine cultures were negative. Arterial blood gas (ABG) analysis revealed respiratory alkalosis with mild compensatory metabolic acidosis as shown in Table 1, and laboratory tests showed a rise in white blood cell count to 15,540 cells/mm^3^ with neutrophils at 82.3% and lymphocytes at 14.9%. CRP levels increased further to 69.67 mg/dL. Both ABG and laboratory findings were consistent with clinical degradation. Complete urine examination (CUE) revealed plenty of red blood cells and 1+ proteinuria.

**Table 1:** Arterial blood gas analysis of the mother HCO_3_^-:^ Bicarbonate; pCO_2_: Partial pressure of carbon dioxide; pO_2_: Partial pressure of oxygen

Parameters	Observed valve	Reference range
pH	7.52	7.35 - 7.45
pCO_2_	24	35 - 45 mm Hg
pO_2_	71	80 - 100 mm Hg
HCO_3_^-^	19.6	22 - 29 mmol/L
Base excess	-2.2	-2.0 - 3.0 mmol/L
Lactic acid	0.9	0.5 - 2.2 mmol/L

**Figure 1:**
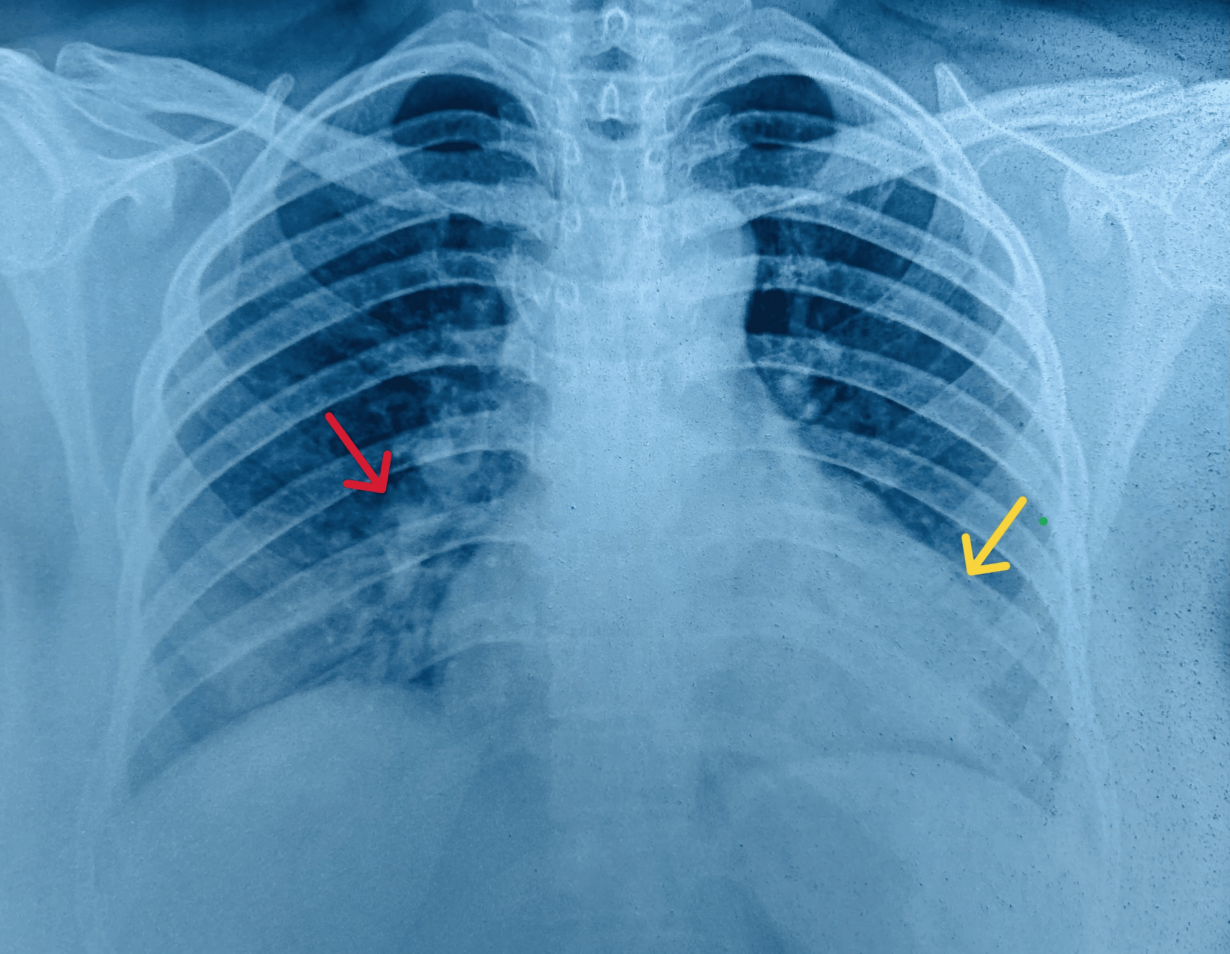
Chest radiograph showing opacities (red arrow) and cardiomegaly (yellow arrow)

On postoperative day 4, chest radiograph showed persistent bilateral basal and right middle lobe opacities with cardiomegaly (Figure 1). CRP levels had decreased to 28.64 mg/L. Over the next four days, her respiratory status gradually improved, and oxygen support was weaned off. She was discharged in stable condition.

**Figure 2:**
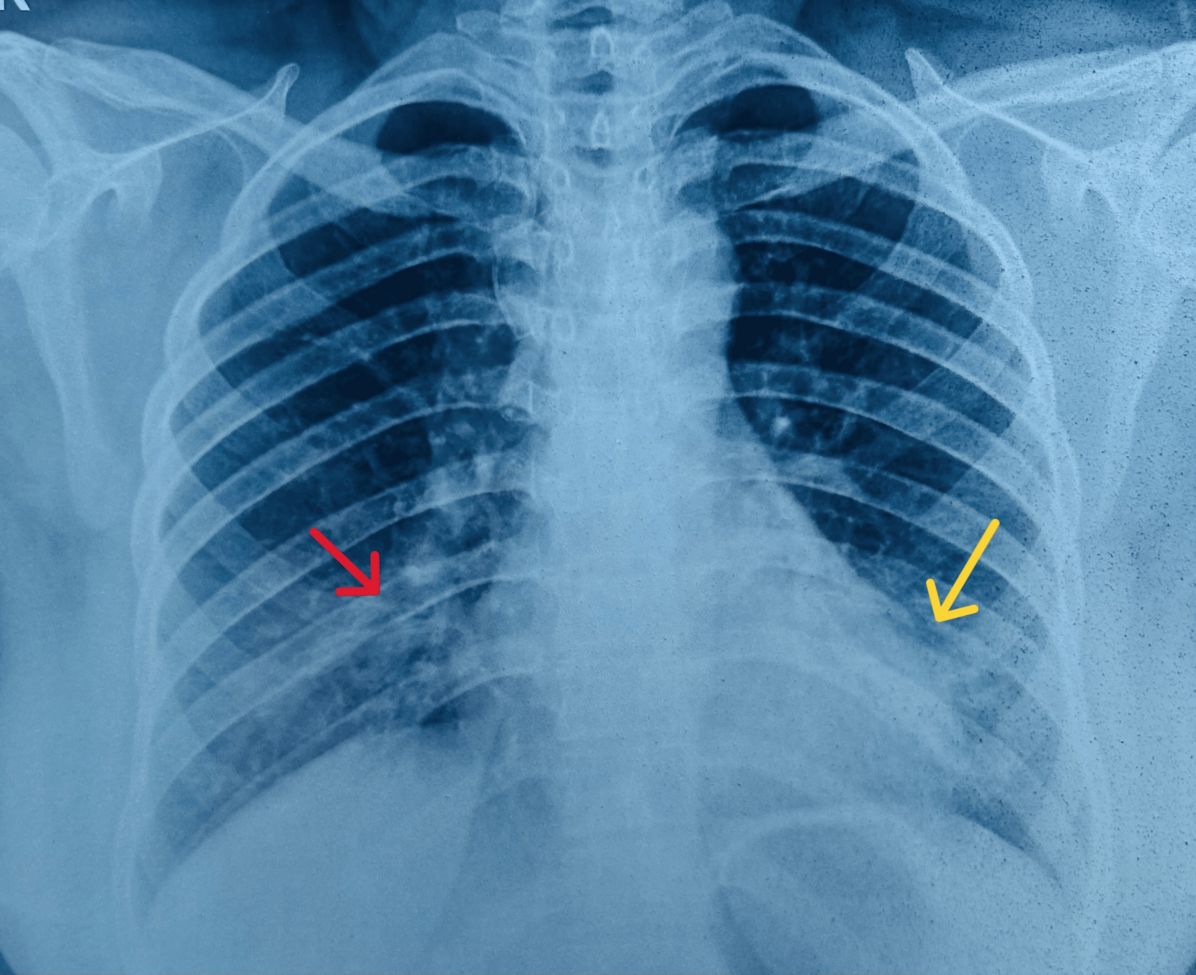
Chest radiograph showing resolving opacities (red arrow) and reduction in cardiomegaly (yellow arrow)

At follow-up, the patient reported occasional cough and mild exertional dyspnea. Chest radiograph on postoperative day 11 showed resolving right middle lobe and bilateral basal opacities with reduced cardiomegaly (Figure 2). CRP levels had declined to 12 mg/dL, reflecting resolution of the inflammatory process. The initial postoperative urine analysis demonstrated 1+ proteinuria despite the absence of clinical signs or laboratory findings indicative of preeclampsia. Repeat urine analysis at follow-up showed no proteinuria, effectively excluding it. The significant maternal laboratory values over the due course are summarized in Table 2.

**Table 2:** Comparative summary of significant maternal laboratory values WBC: White blood cell count; CRP: C-reactive protein

Day	WBC (reference range 4,000 - 10,000 cells/mm^3^)	Neutrophils (reference range 40 - 80%)	Lymphocytes (reference range 20 - 40%)	CRP (reference range < 5 mg/dL)
Preoperative (at presentation)	7,360	77.4	10.3	7.03
Postoperative day 1	6,140	70.6	21.2	63
Postoperative day 2	15,540	82.3	14.9	69.67
Postoperative day 4	_	_	_	28.64
Postoperative day 11	7,820	63.5	24.1	12

The neonate was clinically stable, active, tolerating feeds well at follow-up with no significant weight loss and no signs of respiratory distress, and maintained adequate urine and stool output. Neurological status was unremarkable, and growth parameters were within acceptable limits.

## Discussion

This case report emphasizes the importance of maintaining a high index of suspicion for cardiopulmonary decompensation in the postpartum period, with mild LRTI in the peripartum period, especially in those patients with risk factors like obesity and subclinical cardiac conditions.

Although the maternal presentation symptoms - subjective fever, dry cough, and mild dyspnea - were not alarming, the presence of maternal relative tachycardia, fetal tachycardia, and mildly elevated CRP (7.03 mg/dL) levels, which potentially indicate systemic inflammation, suggested possible progression of LRTI. Given the maternal condition and the non-reassuring fetal heart rate pattern, a multidisciplinary team deemed it appropriate to proceed with delivery via LSCS under spinal anesthesia. This collaborative approach underscores the importance of balancing maternal and fetal risks in late preterm deliveries.

The decision to withhold antenatal corticosteroids aligns with current guidelines of the WHO, which recommend risk-benefit analysis before administration. In this case, there were no imminent signs of preterm labor [6].

Postoperatively, the patient's increased dyspnea, transition from dry to productive cough, desaturation, along with elevated CRP levels and radiographic findings of consolidations in bilateral basal zones and the right middle lobe, established the diagnosis of pneumonia. In addition to the infectious etiology, superimposed factors such as obesity-related respiratory mechanics and postoperative incisional pain, which may lead to shallow breathing, likely contributed to respiratory compromise. The abrupt withdrawal of placental hormones following delivery, which normally sustains a low-resistance vascular circuit during pregnancy, leads to an increase in systemic vascular resistance and afterload. Additionally, the autotransfusion of uterine blood after involution and venacaval pressure release elevates preload [7]. These hemodynamic changes, compounded with postoperative intravenous fluid administration, transiently burden the maternal heart. These changes are normally compensated by a healthy woman, but in our case, the presence of mild underlying cardiac dysfunction may have contributed to the progression of cardiac dysfunction to grade II [8], potentially leading to pulmonary congestion. In the setting of pneumonia, this could have further exacerbated her respiratory status. 

Obesity, as a proinflammatory state and immune disruptor, serves as an additional compounding factor in the setting of pneumonia, potentially contributing to delayed recovery, as observed in our case [9]. While the existing literature documents maternal obesity as a risk factor for gestational hypertension, preeclampsia, gestational diabetes mellitus (GDM), thromboembolic events, preterm delivery, and intrauterine fetal growth restriction [10], there is limited understanding of how obesity influences maternal outcomes during the early postpartum period, in the context of preexisting respiratory condition or infections, underscoring the need for increased clinical and research focus in this domain. However, addressing weight management in the preconception and antenatal period can significantly reduce maternal and fetal risks. The American College of Obstetricians and Gynecologists (ACOG) recommends that women attain a normal BMI before attempting pregnancy, emphasizing dietary modifications and at least 150 minutes of moderate exercise per week [11]. Additionally, ACOG endorses the Institute of Medicine (IOM) guidelines, which recommend a gestational weight gain of 5-9.1 kg for singleton pregnancies in women with obesity [12].

Despite a previous history of preeclampsia, our patient remained normotensive throughout the current pregnancy, with no proteinuria, organ dysfunction, or abnormal findings on uteroplacental Doppler assessment, effectively excluding a recurrence. However, literature indicates that the effects of preeclampsia extend beyond the affected pregnancy. Persistent endothelial dysfunction post-preeclampsia has been attributed to factors such as impaired endothelium-mediated vasodilation, heightened angiotensin II sensitivity, and endothelin receptor subtype B dysregulation, as outlined in a 2018 review [13]. The subclinical grade I diastolic dysfunction observed in our patient may be attributable to her past history of preeclampsia, as a 2019 systematic review has shown that women with prior preeclampsia exhibit cardiac structural changes and impaired diastolic function, which may predispose them to future cardiovascular disease [14].

This case report also illustrates the clinical presentation and management of a late preterm neonate who developed early respiratory distress and was diagnosed with early-onset neonatal sepsis (EOS) and congenital pneumonia, supported by clinical findings and radiographic evidence of bilateral lung infiltrates. The heightened risk of neonatal respiratory morbidity may be attributed to the absence of antenatal steroid coverage and lack of physiological lung compression, disrupted maternal-fetal hormonal transitions associated with caesarean delivery [15], and a perinatal infectious source. The diagnosis of EOS in this case is supported by the National Institute for Health and Care Excellence (NICE) CG195 guidelines, which recommend empirical antibiotic therapy in neonates presenting with either one red flag or two or more non-red flag clinical indicators or risk factors [16]. This neonate had multiple non-red flag clinical indicators, which were signs of respiratory distress (tachypnea, nasal flaring, subcostal retractions, grunting), abnormal heart rate (tachycardia), hypoxia (desaturation), and altered glucose homeostasis (hypoglycemia), fulfilling the criteria for prompt empirical therapy. Although blood and placental cultures were negative, the clinical course marked by worsening distress requiring higher respiratory support and radiological findings and progressive improvement on treatment initiation justified the diagnosis and treatment, emphasizing that culture-negative sepsis remains a well-recognized entity in neonatal care, where timely clinical judgment and intervention are essential to improve outcomes.

Watchful monitoring of the postpartum period enables appropriate and timely interventions of antimicrobials, low-flow oxygen, diuretics, thromboprophylaxis, and cautious intravenous fluids management in the mother, likely averting further complications such as acute respiratory distress syndrome (ARDS), heart failure, or thromboembolic events, and potentially avoiding admission to intensive care unit (ICU). Similarly, early identification of the neonate’s risk for vertical transmission of infection prior to delivery allows for strategic management of anticipated respiratory complications.

## Conclusions

This dual case report highlights the complex interplay between obesity, respiratory infection, cardiac dysfunction, and postpartum physiological shifts contributing to maternal deterioration and also the risk of vertical transmission of maternal infection leading to neonatal complications such as congenital pneumonia. The favourable maternal and neonatal outcomes in this case were achieved through timely multidisciplinary decision-making, vigilant postoperative monitoring, and proactive neonatal care, reinforcing the importance of risk stratification in high-risk pregnancies. Importantly, the report also emphasizes the need to integrate obesity management into preconception and routine antenatal care to reduce the burden of complications. It further supports considering routine antenatal echocardiographic screening, particularly in women with a prior history of preeclampsia, irrespective of its recurrence, which may help identify subclinical cardiac abnormalities and enable tailored care.
